# Psychometric Properties of the German Version of the Pain Vigilance and Awareness Questionnaire (PVAQ) in Pain-Free Samples and Samples with Acute and Chronic Pain

**DOI:** 10.1007/s12529-016-9585-4

**Published:** 2016-08-01

**Authors:** M. Kunz, E. S. Capito, C. Horn-Hofmann, C. Baum, J. Scheel, A. J. Karmann, J. A. Priebe, S. Lautenbacher

**Affiliations:** 1 Physiological Psychology, University of Bamberg, Bamberg, Germany; 2 Department of Family Medicine, Geriatrics Section, University Medical Center Groningen, Groningen, the Netherlands; 3 Psychology School, Hochschule Fresenius University of Applied Sciences, Frankfurt, Germany

**Keywords:** Pain Vigilance and Awareness Questionnaire, Attention to pain, Hypervigilance, psychometric quality

## Abstract

**Purpose:**

The way individuals attend to pain is known to have a considerable impact on the experience and chronification of pain. One method to assess the habitual “attention to pain” is the Pain Vigilance and Awareness Questionnaire (PVAQ). With the present study, we aimed to test the psychometric properties of the German version of the PVAQ across pain-free samples and across patients with acute and chronic pain.

**Method:**

Two samples of pain-free individuals (student sample (*N* = 255)/non-student sample (*N* = 362)) and two clinical pain samples (acute pain patients (*N* = 105)/chronic pain patients (*N* = 36)) were included in this cross-sectional evaluation of the German PVAQ. Factor structure was assessed using exploratory and confirmatory factor analyses. Reliability was assessed using internal consistency (Cronbach’s alpha). Construct validity was tested by assessing correlations between PVAQ and theoretically related constructs.

**Results:**

Exploratory factor analysis (non-student sample) and confirmatory factor analysis (student sample, acute pain patient sample) suggested that a two-factor solution best fitted our data (“attention to pain,” “attention to changes in pain”). Internal consistency ranged from acceptable to good in all four samples. As hypothesized, the PVAQ correlated significantly with theoretically related constructs in all four samples, suggesting good construct validity in pain-free individuals and in pain patients.

**Conclusion:**

The German PVAQ shows good psychometric properties across samples of pain-free individuals and patients suffering from pain that are comparable to PVAQ versions of other languages. Thus, the German PVAQ seems to be a measure of pain vigilance equally valid as found in other countries.

## Introduction

It is well known that the way a person is attending to pain can have a direct effect not only on the experience of pain at that given moment but might also contribute to the development of chronic pain [1–3]. Moreover, pain patients who are highly attentive to pain have been found to engage in fewer productive activities and report greater levels of distress, disability, anxiety, and depression [1]. Thus, a person’s degree of attention to pain is of great clinical relevance.

In order to assess the habitual “attention to pain,” McCracken developed the Pain Vigilance and Awareness Questionnaire (PVAQ [1]). The original English version is a 16-item self-report questionnaire measuring the frequency of self-monitored and self-reported attentional habits with the focus on pain and changes in pain over the past 2 weeks. The psychometric properties of the PVAQ were first tested in a sample of chronic low back pain patients, with satisfactory reliability, good internal consistency, and good validity outcomes [1]. Meanwhile, the PVAQ has been validated in various clinical and nonclinical samples (e.g., [4, 5]) and has been translated into Dutch [6], Chinese [2], Spanish [7], and Italian [8].

With regard to the factorial structure of the PVAQ, the findings are slightly inconsistent. McWilliams and Asmundsons’ [5] were the first to assess the factor structure of the original PVAQ and found a three-factor structure, accounting for 63 % of the variance. The three factors were labeled as “awareness of change,” “monitoring,” and “intrusion.” However, in subsequent studies, a two-factor structure was favored. Roelofs et al. [6] used exploratory factor analysis with oblique rotation on a Dutch pain-free student sample and yielded a two-factor solution, which was asserted in a second sample by confirmatory factor analysis. The first factor was labeled attention to pain, and the second factor was labeled “attention to changes in pain.” This two-factor structure was then replicated in an American and a Dutch sample [9]. Moreover, comparable two-factor structures were also found for shortened versions of the PVAQ tested in American [4] (13 items), Chinese [2] (13 items), Spanish [7] (9 items), and Italian samples [8] (13 items) of chronic pain patients. Thus, most studies support the two-factor structure of the PVAQ. Nevertheless, although clearly favoring the two-structure solutions, several studies also found satisfactory goodness-of-fit for the originally proposed three-structure solution of the PVAQ [6, 7, 9]. Besides investigating the factorial validity, most studies also assessed the construct validity of the PVAQ by correlating the scale with other self-report questionnaires addressing similar pain- or vigilance-related constructs. For this purpose, several authors used the Fear of Pain Questionnaire (FPQ [10]), Pain Anxiety Symptom Scale (PASS [11]), the Pain Catastrophizing Scale (PCS [12]), the Tampa Scale of Kinesiophobia (TSK [13]), and the Hospital Anxiety and Depression Score (HADS [14]). As expected, the PVAQ showed moderate to high positive correlations especially with pain-related self-report questionnaires (PCS, PASS, FPQ) [2, 6–8].

The relevance of using the PVAQ in clinical as well as experimental settings has also been shown in German research studies. In 2009, Lautenbacher and colleagues translated the PVAQ into German. Using this (at that time) non-validated version of the PVAQ, close correlations with pain sensitivity (pain thresholds) [15] were shown as well as a positive prediction of postoperative pain [16, 17]. Despite of these promising findings, further validation of the German version is necessary. This was the aim of the present study.

The PVAQ has been validated in clinical and non-clinical samples. Non-clinical samples were composed of pain-free student samples [5, 6], whereas clinical samples were composed of patients suffering from (chronic) pain [2, 4, 7, 8]. These non-clinical and clinical samples did not only differ with regard to pain, but often also with regard to age, education, and occupation. Whereas student samples are quite homogenous with a similar level of education and a small age range, pain patient samples usually included a broader variety of occupations, educational levels, and age. In order to bridge this gap between non-clinical and clinical samples, we decided to test the psychometric properties of the German PVAQ in four different samples: *young pain-free students; young patients suffering from acute postoperative pain* (who are comparable to the pain-free student sample with regard to age, education, and occupation), *middle-aged pain-free non-students* (with participants being more comparable to chronic pain patients with regard to age, education, and occupation), and *middle-aged patients suffering from chronic pain*. Based on previous findings, we expect that psychometric properties of the PVAQ should be comparable across pain-free and pain patient samples [6, 9].

To test the psychometric properties of the German PVAQ, we first conducted an exploratory factor analysis in the middle-aged pain-free non-student sample. This sample can be seen as the connecting link between previously studied samples of healthy students [6] and chronic pain patients [2, 7–9]. Second, to test the stability and generalizability of the factor structure obtained in the exploratory factor analysis, confirmatory factor analyses were conducted in all other samples large enough for this type of analysis, i.e., young pain-free individuals (pain-free student sample) as well as in a sample of young patients suffering from acute postoperative pain (funnel chest correction). Third, floor/ceiling effects as well as internal consistency were assessed in the two pain-free samples (students and non-students) as well as in the two pain patient samples (acute postoperative pain and chronic pain). In a last step, construct validity was tested using correlations between PVAQ and other pain-related self-report questionnaires (PCS, PASS) (in all four samples) as well as between PVAQ and pain ratings and depression scores in the two pain patient samples.\.

## Methods

This cross-sectional study was conducted in four different samples: two samples of pain-free individuals and two samples of patients suffering from acute or chronic pain. Across the four different samples, the procedure of filling out the questionnaires was kept stable. Questionnaires were always filled out in a single block (not separated by other tasks) under the supervision of the investigator. The study protocols were all approved by the ethics committee of the University of Bamberg. Informed written consent was obtained from all individual participants included in the study.

### Participants

#### Pain-Free Individuals

After translating the PVAQ into German in 2009, we used this questionnaire in several investigations conducted in our laboratory. For validation purpose, we now selected all those investigations, in which the PVAQ was related to the Pain Catastrophizing Scale (PCS) and the Pain Anxiety Symptom Scale (PASS) in order to be able to compute construct validity testing. This selection [15, 17–26] resulted in a sample size of 617 healthy pain-free participants. Participants were recruited via advertisements posted in the university building or via advertisements in the local newspaper (Bamberg). Exclusion criteria were always acute or chronic pain and mental disorders in the last 10 years. The sample of 617 participants was splitted into two sub-samples, namely into a non-student sample and a university student sample. General characteristics of the two samples are displayed in Table 1. Whereas all participants of student sample were enrolled at our University and had obtained a higher education entrance qualification before (Abitur), the educational background, occupation, and age of the non-student sample were much more diverse (see Table 1).

**Table 1:** Descriptive characteristics of the four samples included in the study

Samples		Pain-free individuals		Pain patients	
		Middle-aged non-students	Young students	Middle-aged chronic pain patients	Young acute pain patients
*N*		362	255	38	105
Sex (male/female %)		45/55	48/52	42/58	100/0
Age (mean, SD) in years		41.8 (12.6)	22.8 (3.8)	46.2 (8.7)	19.3 (5.0)
Education (%)	High school not completed	1	0	3	0
	Lower secondary school (Hauptschule)	11	0	47	7
	Intermediate secondary school (Realschule)	32	0	34	20
	Higher education entrance qualification (Abitur) (finished or enrolled)	30	100	8	73
	University degree	26	0	8	0
Employment (%)	Student	0	100	0	79
	Employed	74	0	83	21
	Housewife/housemen/unemployed	26	0	17	0
Questionnaires (mean (SD))	PVAQ	35.2 (11.3)	35.0 (10.5)	46.2 (10.4)	39.2 (9.8)
	PCS	13.6 (8.4)	20.5 (7.6)	27.4 (10.3)	18.3 (8.2)
	PASS	68.5 (28.6)	87.9 (26.5)	106.2 (36.0)	79.7 (27.1)
Depression (mean (SD))	HADS / CES-D	-	-	9.7 (4.7)^a^	21.2 (7.7)^b^
Pain ratings (mean (SD))	NRS	-	-	6.9 (1.6)^c^	3.9 (2.0)^d^
Use of drugs (%)	Analgesics (total)	0	0	82	100 (PCEA)
	NSAIDS	-	-	61	-
	Opioids	-	-	13	100 (PCEA)
	Antidepressants	-	-	34	-

^a^HADS (range of the scale: 0–21; scores >8 indicating potential risk for clinical depression)

^b^CES-D (range of the scale: 0–60; scores >16 indicating potential risk for clinical depression)

^c^Four preceeding weeks

^d^Last week after surgery

*PVAQ* Pain Vigilance and Awareness Questionnaire, *PCS* Pain Catastrophizing Scale, *PASS* Pain Anxiety Symptom Scale, *NRS* Numerical Rating Scale, *CES-D* Center for Epidemiological Studies Depression Scale, *HADS* Hospital anxiety and Depression Score, *NSAIDs* nonsteroidal anti-inflammatory drugs

#### Clinical Pain Samples

We included two clinical pain samples.

The acute pain sample was composed of 105 young, male patients with surgical corrections of congenital malformations of the thorax (funnel chest) (acute postoperative sample, see Table 1). Most of these patients were students and with regard to age comparable to the pain-free student sample. They were recruited among inpatients of the Department of Pediatric Surgery of the University of Erlangen. This department is specialized for the surgical correction of thorax malformations, and this surgical technique, the so-called Erlangen technique of funnel chest correction, is described in detail in Weber and Hümmer [27]. Patients were tested 1 week after the surgery took place, while they were still in the hospital but immediately before discharge. In the week after surgery, the patients were treated by a standard analgesic regimen described elsewhere in detail [16, 17] and still experienced postoperative pain at the time they participated. Exclusion criteria were preoperative pain and severe mental disorders.

The second clinical sample was composed of 38 chronic pain patients suffering either from musculoskeletal back pain (83 %) or from fibromyalgia (17 %). With regard to age, they are comparable to the non-student pain-free sample (see Table 1 for more details). Chronic pain patients were recruited among outpatients attending a multimodal 4-week pain management program at the outpatient unit for pain therapy of the Sozialstiftung Bamberg (Bamberg, Germany). We included patients with predominant musculoskeletal pain (neck pain, upper back pain, low back pain, or fibromyalgia) with pain lasting for a minimum of 6 months. Headaches (migraine, tension-type headache, or non-specified headache) were allowed as secondary diagnosis. Exclusion criteria were surgical interventions within the last year and severe mental disorders.

### Measures

#### *PVAQ*—Assessed in All Samples

The original version of the PVAQ [1] is a 16-item self-report questionnaire. Items are registered on a six-point scale, ranging from 0 (never) to 5 (always); thus, the total questionnaire scores range between 0 and 80. The PVAQ measures the frequency of self-monitored and self-reported attentional habits with the focus on pain and changes in pain over the past 2 weeks. Previous studies have reported good reliability and validity values for the PVAQ [6].

##### Adaption Process of the PVAQ

Adaption of the PVAQ was done following the principles of good practice for the translation and cultural adaptation process for patient-reported outcome (PRO) measures based on the report of the ISPOR task force [28].

In a first step, the 16 items were translated into German by two German native speakers (a professional translator and a pain expert) independently of each other. Both translators were informed by the principal investigator (SL) about the concept of the PVAQ beforehand. In case of discrepancies between translators, these were discussed between the two translators, and a compromise was found for each case. In a second step, an English native speaker back-translated these items into English (a teacher at the Language Center at the University of Bamberg, who supervised the whole translation process). The principal investigator (SL) reviewed the back-translation together with the English native speaker, and translation accuracy was assumed to be sufficient when the English native speaker’s translation was equivalent (in content and concept) with the original version of the PVAQ.

#### *PCS—*Assessed in All Samples

The Pain Catastrophizing Scale (PCS) [29] (German version [30]) was developed as a measure of catastrophic thinking related to pain. Participants are instructed to reflect on thoughts or feelings during past painful experiences. The scale contains 13 items that are rated on a five-point scale, with the end points “not at all” and “all the time.” The PCS has widely been widely used in research on pain catastrophizing and has been shown to have high internal consistency [12, 29] and good validity [30]. Cronbach’s alpha values obtained in the present samples ranged between 0.84 and 0.9, thus indicating good to excellent reliability.

#### *PASS—*Assessed in All Samples

The Pain Anxiety Symptom Scale (PASS) [11] (German version [31]) is composed of four subscales: cognitive anxiety, escape/avoidance, fearful appraisal, and physiological anxiety, and is designed to measure pain anxiety across cognitive, behavioral, and physiological domains. The 40 items are rated on a 6-point scale, with the end points “never” and “always.” The PASS has been shown to have high internal consistency [11, 31] and good validity [31]. Cronbach’s alpha values obtained in the present samples ranged between 0.91 and 0.93, thus indicating excellent reliability.

#### *NRS—*Assessed in the Two Patient Samples

Patients were asked to rate the pain they felt in the last week (acute pain) or in the last 4 weeks (chronic pain), respectively, using a Numerical Rating Scale (NRS) that ranged from 0 (no pain) to 10 (worst pain imaginable).

#### *Depression Scales*—Assessed in the Two Patient Samples

In the acute postoperative sample, depression was assessed using the German version of the Center for Epidemiological Studies Depression Scale (CES-D [32], German version: ADS [33]). The CES-D is a self-rating scale that was designed to assess emotional, somatic, and cognitive symptoms of depressive mood during the last week. It contains 20 items that are rated on a 4-point Likert scale, with end points 0 = rarely or none of the time and 3 = most or almost all the time.

In the chronic pain sample, depression was assessed using the German version of the Hospital anxiety and Depression Score (HADS) [14, 34]. The HADS is a self-rating scale and assesses anxiety (7 items) and depressive symptoms (7 items) that are rated on 4-point Likert scale (verbal descriptors for the Likert scale (0–3) vary depending on the item). For further analysis, we used the SUM score of the depressive symptoms.

Internal consistency and validity for both depression scales have been found to be good [33, 34]. In the present samples, Cronbach’s alpha values (>0.80) also indicated good reliability.

### Statistical Analysis

#### Factor Analyses

##### (I) Exploratory Factor Analysis

An exploratory factor analysis was run in the pain-free non-student sample using principal component analysis with oblique rotation to reveal a suitable number of factors for the German version of the PVAQ. In order to determine the correct number of factors, we applied Parallel Analysis [35], used Kaiser’s eigenvalue >1 criterion [36], and the scree test [37], with the scree-test being considered the most crucial test. We decided to first conduct an exploratory factor analysis, as a first naïve and linear approximation of the German version of the PVAQ (to account for possible language- or culture-related dissimilarities to the original version).

##### (Ii) Confirmatory Factor Analysis

Next, we conducted a confirmatory factor analysis in the pain-free student sample as well as in the acute postoperative patient sample to verify the hypothesized factor structure that we have obtained by exploratory analysis and evaluated its fit. We tested the global model fit via χ2 test. Moreover, we applied the root mean square error of approximation (RMSEA) with its associated 90 % confidence interval (CI), the comparative fit index (CFI), and the non-normed fit index (NNFI) to our data. The following thresholds indicate a good model fit: χ^2^/df < 3, RMSEA ≤ .08, CFI ≥ .90, and NNFI ≥ .90 [38]. We chose the above mentioned fit indexes based on previous studies investigating the factorial structure of the PVAQ [3, 6–8].

### Floor/Ceiling Effects

Floor/ceiling effects were assessed using descriptive statistics and were defined to be present when >15 % of the participants obtained the lowest (0) or highest (80) possible score on the PVAQ.

### Reliability

As an indicator of reliability, we assessed internal consistency using Cronbach’s alpha. It is suggested that values >.9 represent excellent, values >.8 good, values >.7 acceptable, and values <.7 represent questionable or poor reliability [39].

### Construct Validity

With regard to construct validity, we hypothesized a priori that the PVAQ and its subscales would be significantly associated with related constructs. More precisely, given previous findings [6–9], we expected moderate to substantial positive correlation with the Pain Catastrophizing Scale (PCS) and the Pain Anxiety Symptom Scale (PASS) in pain-free individuals as well as in pain patients. Moreover, also based on previous findings [2, 6–9], we expected low positive correlations with clinical pain intensity ratings and with depression in the two patient samples.

Correlation coefficients were interpreted as follows: strong (*r* ≥ .70), moderate to substantial (.30 < *r* < .70), weak (*r* ≤ .30). If more than 75 % of the hypotheses are confirmed, construct validity will be considered as good [8].

The alpha-level was set to 0.05.

We used the IBM Statistical Package for Social Sciences (SPSS version 23 for Windows and SPSS Amos 23) to conduct all analyses.

## Results

### Descriptive Statistics (All Four Samples)

Table 1 presents descriptive information on the four samples tested.

With regard to PVAQ scores, we found a significant difference between samples (Kruskal-Wallis test: χ^2^ = 45.6, *p* < 0.001). Using Mann-Whitney *U* tests to compare each sample with another, we found that PVAQ scores were significantly elevated in the two patient samples compared to the pain-free samples (all *p* values <0.001). Moreover, chronic pain patients obtained higher PVAQ scores than the acute pain patients (*p* < 0.001). No difference in PVAQ scores was found between the two pain-free samples (*p* = 0.575).

With regard to the PCS and the PASS scales, we also found significant group differences (Kruskal-Wallis test, both *p* values <0.001). Mann-Whitney *U* tests revealed significant differences between all samples for both scales (all *p* values <0.05). As can be seen in Table 1, chronic pain patients scored highest on the PCS and PASS scales whereas the pain-free non-student sample obtained the lowest scores. Surprisingly, the pain-free student sample scored higher on the PASS and the PCS scale than the acute pain sample.

With regard to the pain experienced by the pain patients, chronic pain patients reported significantly higher pain intensities compared to the acute pain patients (Mann-Whitney *U* tests, *p* < 0.001).

### Factor Analyses

#### (I) Exploratory Factor Analysis (Pain-Free Non-student Sample)

Eigenvalues >1 criterion and the parallel analysis proposed solutions between one to three factors. The scree test (most crucial test) indicated that indeed a two-factor solution is best fitting for our data set. This two-factor solution accounted for 43.7 % of the variance. The two-factor solution is displayed in Table 2. Given that our two-factor structure is identical to the two-factor structure reported by Roelofs et al. [6], we used the same labeling as suggested previously and labeled our first factor “attention to changes in pain” and the second “attention to pain.”

**Table 2:** Factor loadings of the PVAQ as obtained by exploratory factor analysis (pain-free non-student sample)

Items	Two-factor solution	
	attention to changes in pain	attention to pain
3. I am quick to notice changes in pain intensity.	−.815	.068
9. I know immediately when pain starts or increases.	−.544	.314
2. I am aware of sudden or temporary changes in pain.	−.676	−.057
5. I am quick to notice changes in location or extent of pain.	−.796	.173
11. I know immediately when pain decreases.	−.585	−.081
4. I am quick to notice effects of medication on pain.	−.638	.073
14. I keep track of my pain level.	−.192	.747
10. When I do something that increases pain. The first thing I do is check to see how much pain was increased.	*−.036*	.613
13. I pay close attention to pain.	−.145	.782
12. I seem to be more conscious of pain than others.	−.083	.629
15. I become preoccupied with pain.	.174	.560
6. I focus on sensations of pain.	−.274	.644
8.* I find it easy to ignore pain.	.175	.457
7. I notice pain even if I am busy with other activity.	−.305	.484
1. I am very sensitive to pain.	−.181	.422
16.* I do not dwell on pain.	.281	.323

*Boldface print indicates salient loadings (≥ .32)*

*Items are reverse scored

#### (Ii) Confirmatory Factor Analyses (Pain-Free Student Sample/Acute Pain Patient Sample)

**Fig. 1:**
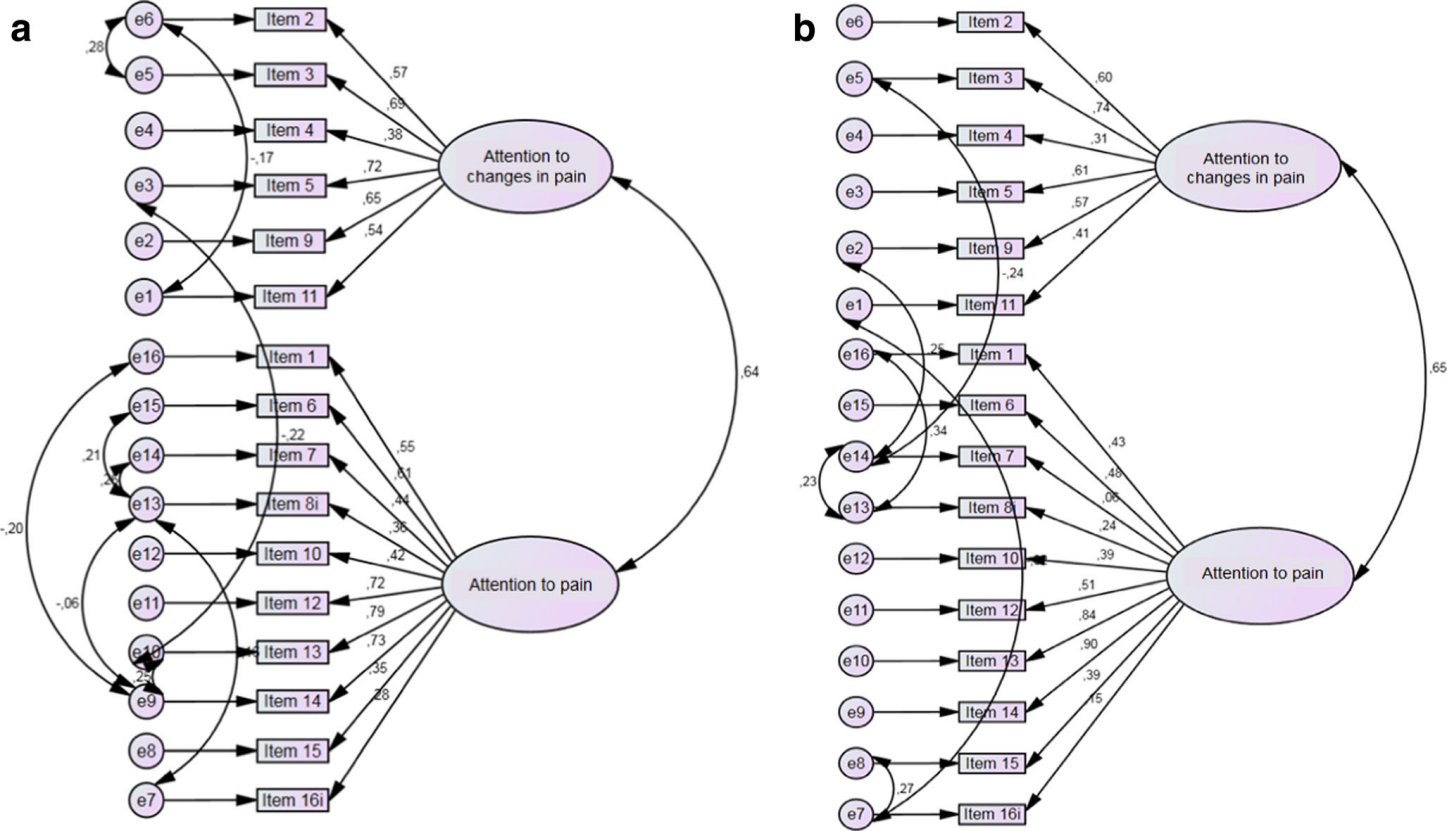
Confirmatory factor analysis - Diagrams of the 2-factor solution (obtained from the exploratory factor analysis) in (a) the pain-free student sample and (b) the acute pain patient sample, with standardized factor loadings of each PVAQ item and commonalities and correlation values specified

The stability of this two-factor structure obtained by exploratory factor analysis was tested using confirmatory factor analysis in the pain-free student sample as well as in the acute pain patient sample (factor analysis could not be conducted in the chronic pain patients, given the small sample size). The goodness-of-fit indices for both samples are displayed in Table 3. The different goodness-of-fit indices indicated good model fits for the two-factor solution in both samples. Only the NNFI value in the acute pain patient sample lies slightly below the good-fit threshold of .90. Figure 1a, b shows the diagrams of the two-factor model (“attention to changes in pain,” “attention to pain”) including the standardized factor loadings of each PVAQ item.

**Table 3:** Confirmatory factor analysis of the PVAQ in pain-free individuals (student sample) and in pain patients (acute pain patient sample, postoperative pain after thorax correction). The Table lists goodness-of-fit indices for the two-factor solution in both samples

Model	χ^2^	df	χ^2^/df	RMSEA	NNFI	CFI
Pain-free students	88.43	82	1.08	0.02	0.99	0.99
Acute pain patients	131.51	92	1.43	0.06	0.88	0.91

*PVAQ* Pain Vigilance and Awareness Questionnaire,

*χ* ^*2*^ */df* Chi-square divided by degrees of freedom, *RMSEA* root mean square error of approximation, *NFI* non-normed fit index, *CFI* comparative fit index

### Correlation Between the PVAQ and Its Subscales (All Four Samples)

The PVAQ sum scores correlated strongly with the two subscales in all samples tested, with correlation coefficients ranging between 0.74 and 0.78 (“attention to changes in pain”) and 0.88 and 0.91 (“attention to pain”), respectively. In contrast, the two subscales correlated only moderately with each other (correlation coefficients ranging between 0.33 and 0.51).

### Floor/Ceiling Effects (All Four Samples)

No floor or ceiling effect was found in any of the four samples, since 0 % of the participants obtained the lowest (0) or highest (80) possible score on the PVAQ.

### Reliability (All Four Samples)

Cronbach’s alpha values of the PVAQ were computed separately for the four samples tested. Cronbach’s alpha values for the whole PVAQ ranged between 0.77 and 0.84 and thus, indicated acceptable too good reliability. With regard to Cronbach’s alpha values for the two subscales, these ranged between 0.72 and 0.80 (“attention to changes in pain”) and 0.73 and 0.79 (“attention to pain”), respectively, thus indicating acceptable to good reliability also for the subscales of the PVAQ.

### Construct Validity

#### (I) Related Constructs (PCS, PASS) (All Four Samples)

Pearson’s correlations were computed between PVAQ (total sum score and subscale scores of the two-factor solution) and the other pain-related questionnaires (total sum scores). The results of all correlation analyses are displayed in Table 4. As hypothesized, the PVAQ total score showed moderate to substantial positive associations with theoretically related constructs, namely pain catastrophizing and pain-related anxiety, across all four samples. In accordance with previous studies [9], we found that the PVAQ subscale “attention to pain” was stronger related to PCS and PASS scores compared to the subscale “attention to changes in pain” (see Table 4). In line with this, correlations between the subscale “attention to pain” and the two scales PCS and PASS always reached significance in all samples tested, whereas the subscale “attention to changes in pain” only correlated significantly with PCS and PASS in 50 % of the samples studied.

**Table 4:** Construct validity—Pearson correlations of PVAQ (total and its subscales) with PCS and PASS (all four samples) and with pain ratings and depression scores (the two patient samples)

Measure	Sample	PVAQ_total_ (*r*)	PVAQ_subscales_ (*r*)	
			Attention to changes in pain	Attention to pain
PCS	Pain-free non-students	0.37***	0.09	0.47***
	Pain-free students	0.43***	0.21**	0.44***
	Acute pain patients	0.48***	0.27**	0.49***
	Chronic pain patients	0.54***	0.19	0.61***
PASS	Pain-free non-students	0.31***	0.10	0.38***
	Pain-free students	0.50***	0.28***	0.54***
	Acute pain patients	0.61***	0.35***	0.62***
	Chronic pain patients	0.47**	−0.11	0.70***
NRS	Acute pain patients	0.38***	0.17	0.46***
	Chronic pain patients	0.25	0.22	0.19
Depression	Acute pain patients (CES-D)	0.37***	0.12	0.44***
	Chronic pain patients (HADS)	0.05	−0.28	0.27

*PVAQ* Pain Vigilance and Awareness Questionnaire, *PASS* Pain Anxiety Symptom Scale, *PCS* Pain Catastrophizing Scale, *NRS* Numerical Rating Scale; *CES-D* Center for Epidemiological Studies Depression Scale, *HADS* Hospital anxiety and Depression Score

***p < .001; **p < .01; strength of correlation: strong (*r* ≥ .70); moderate to substantial (.30 < *r* < .70); weak (*r* ≤ .30)

#### (Ii) Pain Intensity and Depression (the Two Pain Patient Samples)

Pearson’s correlations were computed between PVAQ (total sum score and subscale scores of the two-factor solution) and pain intensity ratings (NRS scale) as well as depression scores in the two patient samples (see Table 4). As hypothesized, the PVAQ total score showed weak to moderate positive associations with pain intensity ratings of the patients as well as with depression scores. Especially the subscale “attention to pain” showed positive associations with pain intensity ratings and depression scores. Overall correlations in the chronic patients groups tended to be lower and did not reach level of significance compared to the acute pain patient group. Reasons for this are the much smaller sample size in the chronic pain patient group, as well as chronification processes that might complicate the relation between PVAQ and pain intensity as well as depression.

## Discussion

The present study investigated the psychometric properties of the German version of the PVAQ (PVAQ). Although the German version of the 16-item PVAQ was already used in several studies from our lab [15–17, 40], we have not yet systemically elaborated these properties. In order to make the PVAQ available to all German-speaking researchers and clinicians, a validation of the German version was necessary. To do this, we investigated the psychometric properties of the PVAQ in samples of pain-free individuals and in patients suffering from pain using exploratory and confirmatory factor analyses as well as analyses on internal consistency and construct validity.

With regard to the factorial structure of the German version of the PVAQ, we found that a two-factor solution was best suited for our samples. This is in line with findings reported for other language versions of the PVAQ (English, Dutch, Spanish, Chinese, Italian [2, 5–9]) who also found a two-factor solution to be best suited. Furthermore, a high stability of the two-factor solution is also suggested by the fact that the two-factor structure was found suitable across pain-free samples (samples varying with regard to age and educational background) and in patients suffering from acute pain in the present study. This strongly promises that our findings will be generalizable to other samples.

We found the same two-factor solution as originally suggested by Roelofs et al. [6], and thus, we used their labeling “attention to pain” and “attention to changes in pain” for the two factors. McCracken [4] suggested renaming these two factors as “active vigilance” and “passive awareness,” respectively, using a 13-item PVAQ version. The reason McCracken [4] introduced a shortened 13-item PVAQ version was that he found a better model fit after dropping three items of the original PVAQ. However, given that we found good model fits for the original 16-item version, there was no indication to drop any of the 16 items. We thus recommend a 16-item version for the German PVAQ, and we will keep the original labeling of the two factors as suggested by Roeloffs et al. [6]. The German version of the scale and the subscale assignments can be found in the appendix.

We found no floor or ceiling effects in the PVAQ, neither in pain-free individuals nor in patients suffering from acute or chronic pain. Thus, the discriminative power of the German version of the PVAQ is not limited by such effects in clinical as well as in non-clinical samples. Reliability analyses showed acceptable-good internal consistency (Cronbach’s alpha) for the German version of the PVAQ in pain-free samples (0.81/.084) and in patients suffering from pain (0.77/0.79). This is in line with previous findings that indicated Cronbach’s alpha values around 0.8 for other language versions of the PVAQ [6–9].

With regard to the construct validity, we tested the association of the PVAQ (total score and subscale scores) with other conceptually close constructs, namely pain catastrophizing (PCS) and pain anxiety (PASS). As expected, we found positive correlations of moderate to substantial strength between the PVAQ (total score) and PCS as well as the PASS across all four samples studied. Although the two pain patient groups (especially the chronic pain patients) scored higher on the PVAQ compared to the pain-free individuals, similar associations between PVAQ and the other scales were found across pain-free and pain patient samples. Using the (non-validated) German version of the PVAQ in a previous study, we also found significant associations between the PVAQ and the other two scales in cancer patients undergoing surgery [40] (the data from that study were not included in the present study and thus, represent independent evidence). This suggests that individuals who are more vigilant to pain or who focus more on pain also catastrophize more about pain and experience more pain-related fear. Whereas the PVAQ targets the habitual attention to pain and reflects a more cognitive component, PCS and PASS stress more the emotional aspects of pain processing. Here, we would like to make a short reference to the fear-avoidance model, which indeed predicts such relationships between cognitive and emotional aspects of pain processing [42]. With the present study, we could support this assumption of a close relationship between cognitive and emotional aspects of pain processing and show that it can be found across different German samples of pain-free individuals and patients suffering from acute or chronic pain.

In line with previous findings [6–9], we could also show that the subscale “attention to pain” correlated much stronger with PCS and PASS scores compared to the subscale “attention to changes in pain.” Again, this pattern was found consistently across samples studied. This stresses the previous assumption that the subscale “attention to changes in pain” (or passive awareness [4]) might be a unique construct that is not captured in the other pain-related questionnaires [9]. Indeed, the “attention to changes in pain” items require more explicit consideration of temporal features of pain. Is there a change in pain intensity or pain location between at least two time points? Thus, it is necessary to closely monitor pain over a certain period. In most of the other questionnaires targeting cognitive and emotional processing of pain (e.g., PCS, PASS), such a temporal awareness is not necessary in order to answer the questions. Thus, the scale “attention to changes in pain” requires temporal awareness and flexibility and—in comparison to scales of other comparable pain-related questionnaires—might be less a sign of a pathological behavioral pattern. Saying that and considering the moderate correlation between the two subscales of the PVAQ, it becomes clear that both subscales target different aspects of attention to pain. Thus, the separate use of the subscales can be recommended.

Both PCS and PASS are also composed of different subscales (three and four subscales, respectively). Thus, it is possible that different associations between PVAQ and PCS/PASS might be found when looking at single correlations between the different subscales. Although the enormous amount of resulting single correlations dramatically inflates the chance of alpha errors, we also looked at these single correlations. Besides the PASS subscale “cognitive” showing especially strong correlations with the PVAQ (which is in line with the notion that the PVAQ captures more cognitive aspects related to pain), we found that these single correlations yielded no additional information beyond this already revealed by using the SUM scores of PCS/PASS. Therefore, we refrain from reporting these findings in more detail.

In the two pain patient samples, we also computed correlations between PVAQ and pain intensity ratings as well as depression scores. In line with previous findings [3, 4, 8], these associations were lower than those found for PCS and PASS. Finding only weak or no associations between PVAQ and pain intensity ratings is not surprising, given that pain intensity is dependent on various factors (e.g., tissue damage, social factors, affective state, and social environment), with pain vigilance being just one of them.

Our study has some limitations. In contrast to previous translation of the PVAQ (e.g., Italian translation [8]), we did not conduct pilot testing to assess comprehensibility of the German version of the PVAQ (e.g., by use of cognitive interviews). Leaving out this “cognitive debriefing” step, we cannot fully exclude that our translation might include ambiguous phrases [28]. Moreover, we did not assess the retest reliability of the PVAQ in the present study. However, a portion of the acute postoperative pain sample (*N* = 80) took part in a longitudinal study, where we assessed the PVAQ prior to surgery as well as 1 week, 3 months, and 6 months after (funnel chest correction) surgery [41]. As we reported [41], PVAQ scores were relatively stable over time, with correlation values ranging between *r* = 0.56 and 0.62. Thus, the German version of the PVAQ seems to be a consistent and reliable measure of vigilance to pain. Moreover, given the relatively small sample size in the chronic pain patient group, we could not assess the factor structure in this sample. However, given that our two-factor structure of the PVAQ has also been repeatedly found in chronic pain patient groups in other countries, we assume that the two-factor solution of the German version of the PVAQ is also suited for chronic patient groups.

In conclusion, the present data suggest that the psychometric properties of the German version of the PVAQ are very comparable to the original English version of the questionnaire as well as to validated versions of this questionnaire in other languages (Chinese, Dutch, Italian, Spanish). Consequently, the PVAQ appears to be a valid measure of vigilance to pain, which is comparable across cultures. We found a two-factor structure to be best suitable for the German version of the PVAQ, with the factors “attention to pain” (10 items) and “attention to changes in pain” (6 items). The latter subscale seems less associated with pain catastrophizing and pain anxiety, thus potentially forming a unique construct among these pain-related variables reflecting the cognitive and emotional processing of pain.

## Appendix

### PVAQ

Im Folgenden sind 16 Aussagen darüber aufgelistet, wie Leute auf Schmerzen reagieren.

Bitte geben Sie anhand der untenstehenden Skala an, wie häufig jede Aussage auf Sie zutrifft (d.h., wie häufig Sie auf die beschriebene Art und Weise reagieren). Berücksichtigen Sie bei Ihren Bewertungen besonders die letzten zwei Wochen. Halten Sie Ihre Bewertungen fest, indem Sie für jede Aussage eine Zahl zwischen 0 (Niemals) und 5 (Immer) ankreuzen.

Invertierte Items: 8, 16.

Aufmerksamkeit für Schmerzen (Items 1, 6, 7, 8, 10, 12, 13, 14, 15, 16).

Aufmerksamkeit für Schmerzveränderungen (Items 2, 3, 4, 5, 9, 11).
